# The association of coffee intake with liver cancer risk is mediated by biomarkers of inflammation and hepatocellular injury: data from the European Prospective Investigation into Cancer and Nutrition^1^,^2^,^3^

**DOI:** 10.3945/ajcn.115.116095

**Published:** 2015-11-11

**Authors:** Krasimira Aleksandrova, Christina Bamia, Dagmar Drogan, Pagona Lagiou, Antonia Trichopoulou, Mazda Jenab, Veronika Fedirko, Isabelle Romieu, H Bas Bueno-de-Mesquita, Tobias Pischon, Kostas Tsilidis, Kim Overvad, Anne Tjønneland, Marie-Christine Bouton-Ruault, Laure Dossus, Antoine Racine, Rudolf Kaaks, Tilman Kühn, Christos Tsironis, Eleni-Maria Papatesta, George Saitakis, Domenico Palli, Salvatore Panico, Sara Grioni, Rosario Tumino, Paolo Vineis, Petra H Peeters, Elisabete Weiderpass, Marko Lukic, Tonje Braaten, J Ramón Quirós, Leila Luján-Barroso, María-José Sánchez, Maria-Dolores Chilarque, Eva Ardanas, Miren Dorronsoro, Lena Maria Nilsson, Malin Sund, Peter Wallström, Bodil Ohlsson, Kathryn E Bradbury, Kay-Tee Khaw, Nick Wareham, Magdalena Stepien, Talita Duarte-Salles, Nada Assi, Neil Murphy, Marc J Gunter, Elio Riboli, Heiner Boeing, Dimitrios Trichopoulos

**Affiliations:** 4 Department of Epidemiology, German Institute of Human Nutrition Potsdam-Rehbrücke, Nuthetal, Germany;; 5 WHO Collaborating Center for Food and Nutrition Policies, Department of Hygiene, Epidemiology and Medical Statistics, University of Athens Medical School, Athens, Greece;; 6 Hellenic Health Foundation, Athens, Greece;; 7 Department of Epidemiology, Harvard School of Public Health, Boston, MA;; 8 Bureau of Epidemiologic Research, Academy of Athens, Athens, Greece;; 9 International Agency for Research on Cancer, Lyon, France;; 10 Department of Epidemiology, Rollins School of Public Health, Emory University, Atlanta, GA;; 11 Winship Cancer Institute, Emory University, Atlanta, GA;; 12 National Institute for Public Health and the Environment, Bilthoven, Netherlands;; 13 Department of Gastroenterology and Hepatology, University Medical Centre, Utrecht, Netherlands;; 14 Department of Epidemiology and Biostatistics, The School of Public Health, Imperial College London, London, United Kingdom;; 15 Department of Social and Preventive Medicine, Faculty of Medicine, University of Malaya, Kuala Lumpur, Malaysia;; 16 Molecular Epidemiology Group, Max Delbrueck Center for Molecular Medicine, Berlin-Buch, Germany;; 17 Department of Hygiene and Epidemiology, University of Ioannina School of Medicine, University Campus, Ioannina, Greece;; 18 Section for Epidemiology, Department of Public Health, Aarhus University, Aarhus, Denmark;; 19 Diet, Genes and Environment, Danish Cancer Society Research Center, Copenhagen, Denmark;; 20 Inserm, Centre for Research in Epidemiology and Population Health, Nutrition, Hormones and Women's Health Team, Villejuif, France;; 21 University Paris Sud, Villejuif, France;; 22 IGR, Villejuif, France;; 23 Division of Cancer Epidemiology, German Cancer Research Centre, Heidelberg, Germany;; 24 Molecular and Nutritional Epidemiology Unit, Cancer Research and Prevention Institute–ISPO, Florence, Italy;; 25 Department of Clinical and Experimental Medicine–Federico II University, Naples, Italy;; 26 Epidemiology and Prevention Unit, Fondazione IRCCS, Istituto Nazionale dei Tumori, Milan, Italy;; 27 Cancer Registry and Histopathology Unit, “M.P. Arezzo” Hospital, Ragusa, Italy;; 28 HuGeF Foundation, Turin, Italy;; 29 Julius Center for Health Sciences and Primary Care, University Medical Center, Utrecht, Netherlands;; 30 Department of Community Medicine, Faculty of Health Sciences, University of Tromsø, The Arctic University of Norway, Tromsø, Norway;; 31 Department of Research, Cancer Registry of Norway, Oslo, Norway;; 32 Department of Medical Epidemiology and Biostatistics, Karolinska Institutet, Stockholm, Sweden;; 33 Genetic Epidemiology Group, Folkhälsan Research Center, Helsinki, Finland;; 34 Public Health Directorate, Asturias, Oviedo, Spain;; 35 Unit of Nutrition and Cancer, Cancer Epidemiology Research Program, Catalan Institute of Oncology, Bellvitge Biomedical Research Institute, Barcelona, Spain;; 36 CIBER de Epidemiología y Salud Pública, Spain;; 37 Escuela Andaluza de Salud Pública, Instituto de Investigación Biosanitaria ibs.GRANADA, Hospitales Universitarios de Granada/Universidad de Granada, Granada, Spain;; 38 Department of Epidemiology, Murcia Regional Health Authority, IMIB-Arrixaca, Murcia, Spain;; 39 Navarre Public Health Institute, Pamplona, Spain;; 40 Epidemiology and Health Information, Public Health Division of Gipuzkoa, Basque Regional Health Department, San Sebastian, Spain;; 41 Arctic Research Centre, Umeå University, Umeå, Sweden;; 42 Department of Surgical and Perioperative Sciences, Surgery and Public Health, Nutrition Research, Umeå University, Umeå, Sweden;; 43 Department of Clinical Sciences, Lund University, Clinical Research Center, Malmö, Sweden;; 44 Department of Clinical Science, Division of Internal Medicine, Skane University Hospital, Malmö, Lund University, Malmö, Sweden;; 45 Cancer Epidemiology Unit, Nuffield Department of Clinical Medicine, University of Oxford, Oxford, United Kingdom;; 46 Department of Public Health and Primary Care, University of Cambridge, Cambridge, United Kingdom; and; 47 MRC Epidemiology Unit, Institute of Metabolic Science, Addenbrooke’s Hospital, Cambridge, United Kingdom

**Keywords:** biomarkers, coffee, European Prospective Investigation into Cancer and Nutrition, liver cancer, mediation

## Abstract

**Background:** Higher coffee intake has been purportedly related to a lower risk of liver cancer. However, it remains unclear whether this association may be accounted for by specific biological mechanisms.

**Objective:** We aimed to evaluate the potential mediating roles of inflammatory, metabolic, liver injury, and iron metabolism biomarkers on the association between coffee intake and the primary form of liver cancer—hepatocellular carcinoma (HCC).

**Design:** We conducted a prospective nested case-control study within the European Prospective Investigation into Cancer and Nutrition among 125 incident HCC cases matched to 250 controls using an incidence-density sampling procedure. The association of coffee intake with HCC risk was evaluated by using multivariable-adjusted conditional logistic regression that accounted for smoking, alcohol consumption, hepatitis infection, and other established liver cancer risk factors. The mediating effects of 21 biomarkers were evaluated on the basis of percentage changes and associated 95% CIs in the estimated regression coefficients of models with and without adjustment for biomarkers individually and in combination.

**Results:** The multivariable-adjusted RR of having ≥4 cups (600 mL) coffee/d compared with <2 cups (300 mL)/d was 0.25 (95% CI: 0.11, 0.62; *P*-trend = 0.006). A statistically significant attenuation of the association between coffee intake and HCC risk and thereby suspected mediation was confirmed for the inflammatory biomarker IL-6 and for the biomarkers of hepatocellular injury glutamate dehydrogenase, alanine aminotransferase, aspartate aminotransferase (AST), γ-glutamyltransferase (GGT), and total bilirubin, which—in combination—attenuated the regression coefficients by 72% (95% CI: 7%, 239%). Of the investigated biomarkers, IL-6, AST, and GGT produced the highest change in the regression coefficients: 40%, 56%, and 60%, respectively.

**Conclusion:** These data suggest that the inverse association of coffee intake with HCC risk was partly accounted for by biomarkers of inflammation and hepatocellular injury.

## INTRODUCTION

Coffee is among the most frequently consumed beverages around the world. It was long believed that coffee is harmful for health; however, epidemiological data obtained over the last years has provided opposite findings, showing that coffee drinking is inversely associated with the risk of several chronic diseases, including liver disease and primary liver cancer (1, 2). Consequently, 2 recent meta-analyses of 9 case-control and 8 cohort studies reported a 56% (3) and a 50% (4) lower risk of liver cancer for high than for no consumption of coffee. A more recent study within the European Prospective Investigation into Cancer and Nutrition (EPIC) investigated associations between coffee intake and the most common form of primary liver cancer—hepatocellular carcinoma (HCC)^48^ (5). In this large prospective cohort study, participants in the highest quintile of coffee intake had an HCC risk that was ∼70% lower than that of participants with minimal or no consumption (5).

Despite accumulating evidence, the current mechanisms explaining these relations remain unclear. Indeed, it is speculated that the inverse associations reported in epidemiological studies could be accounted for by reduced coffee intake in patients with liver and digestive diseases (3). However, this speculation may not be relevant in prospective analyses involving participants who are apparently healthy at study recruitment. Alternatively, many biologically plausible mechanisms could be implicated in the association between coffee intake and liver cancer. In this vein, coffee has been shown to exert beneficial effects on metabolic-related liver cancer risk factors, such as type 2 diabetes (6) and nonalcoholic fatty liver disease (NAFLD) (7). In addition, coffee was shown to exert anti-inflammatory (8, 9) and hepatoprotective properties (10) and inhibitory effects on hepatocarcinogenesis (11). Finally, coffee has been associated with iron metabolism (12), thereby potentially inhibiting iron-induced liver carcinogenesis (13). On the basis of this evidence, it is conceivable that the inverse association of coffee intake with HCC risk could be mediated, at least in part, through one or more of these biologically plausible mechanisms.

On the basis of data from EPIC, we aimed to evaluate the role of biomarkers representative of different biological processes—metabolic, inflammatory, liver injury, and iron metabolism—as potential mediators in the relation of coffee intake with HCC risk.

## METHODS

### Study population

EPIC was designed to identify nutritional, lifestyle, metabolic, and genetic risk factors for cancer. In brief, in the period 1992–2000, ∼520,000 apparently healthy men and women aged 35–75 y from 10 European countries (Denmark, France, Germany, Greece, Italy, Netherlands, Norway, Spain, Sweden, and the United Kingdom) were enrolled in the study. The study was approved by the Ethical Review Board of the International Agency for Research on Cancer and by the local Ethics Committees in the participating study centers. Participants gave informed consent before enrollment. Procedures were in line with the Helsinki Declaration. At enrollment, standardized questionnaires were used to record sociodemographic, lifestyle, and medical history data. Measurements of weight, height, and waist-to-hip circumferences were performed for most participants. Details of EPIC are given elsewhere (14–16).

### Assessment of coffee intake

Dietary intakes over the previous year were assessed at enrollment through validated study center–specific questionnaires, which also inquired about coffee intake (14). The usual beverage intakes were estimated from the frequency and portion size (in mL). This method was reported to yield very good reliability of coffee consumption compared with repeated 24-h recalls (*r* = 0.70) (17). Total energy intake was calculated as previously reported by using the EPIC Nutrient Database (14).

### Follow-up of study population and case ascertainment

The current analysis is based on a nested case-control study within EPIC using data as of last follow-up of participants in 2006. The median follow-up time from study recruitment to diagnosis of cancer was 7.9 y (IQR: 6.4–9.4 y). HCC was defined as a tumor in the liver per the10th revision of the *International Classification of Diseases*, code C22.0 (18), with morphology codes (8170/3 and 8180/3) based on the *International Classification of Diseases for Oncology* (19)]. The respective histological subtypes, the methods used for the diagnosis of cancer, and α-fetoprotein (AFP) concentrations were reviewed to exclude metastatic cases or other types of liver cancers. After exclusion of cases with other types of cancer before the index case (*n* = 18), metastatic cases (*n* = 23) or cases with ineligible histological subtypes (*n* = 31), 125 HCC cases were identified that occurred over a median of 5 y following recruitment (range: 2.4–6.8 y). With the use of risk set sampling, 2 controls per case were selected at random from all cohort members who had donated a blood sample—who were alive and free of cancer at the time of liver cancer diagnosis of the index case—and were matched to the case on study center, sex, age (±12 mo), date of blood collection (±2 mo), fasting status (<3, 3–6, or >6 h), and time of day (±3 h) at blood collection. Women were additionally matched according to menopausal status [premenopausal, perimenopausal (or unknown), or postmenopausal] and exogenous hormone use (yes, no, or missing) at blood donation.

### Laboratory assays

As described in detail elsewhere (16), blood samples in the EPIC cohort were collected at baseline, processed, divided into heat-sealed straws, and stored in liquid nitrogen at −196°C. Researchers were blinded to the case-control status of the samples. Inflammatory and metabolic biomarkers were measured at the Institute of Clinical Chemistry, University of Magdeburg, Germany (20). C-reactive protein (CRP) was measured by using a high-sensitivity assay (Turbidimetrie; Modular-System) with reagent and calibrators from Roche. IL-6 was measured by electrochemiluminescence immunoassay (Modular-System). C-peptide was measured with an Immulite 2000 (Siemens). Adiponectin, leptin, and fetuin-A concentrations were measured by ELISA (ALPCO Diagnostics; Biovendor; and ALPCO Diagnostics, respectively). To quantify high-molecular-weight (HMW) adiponectin, serum samples were pretreated with a protease that specifically digests low-molecular-weight and HMW adiponectin. Non-HMW adiponectin was calculated by subtracting HMW adiponectin from total adiponectin. Glutamate dehydrogenase (GLDH) was measured on DGKC optimized at 37°C (Modular-System). Liver and chronic hepatitis markers were measured at the Centre de Biologie République. Concentrations of albumin, total bilirubin, alanine aminotransferase (ALT), aspartate aminotransferase (AST), γ-glutamyltransferase (GGT), lactate hydrogenase, and alkaline phosphatase were measured on the ARCHITECT c Systems and the AEROSET System (Abbott Diagnostics) according to standard protocols (21). Hepatitis B surface antigen (HBsAg) and antibodies to hepatitis C virus (HCV) were measured by using ARCHITECT chemiluminescent microparticle immunoassays (Abbott Diagnostics) as described previously (21). Serum iron and transferrin concentrations were measured with a clinical chemical autoanalyzer (Hitachi 912; Roche Diagnostics). The iron metabolism biomarkers have been measured at the International Agency for Research on Cancer. Serum ferritin was measured with a dedicated immunoanalyzer (Access; Beckman). In a pretest study, these biomarkers showed good reliability over several years apart (22). Missing measurements for CRP (*n* = 7), IL-6 (*n* = 105), C-peptide (*n* = 27), adiponectin (*n* = 4), leptin (*n* = 4), GLDH (*n* = 6), fetuin-A (*n* = 6), ALT (*n* = 6), AST (*n* = 6), albumin (*n* = 6), AFP (*n* = 13), total bilirubin (*n* = 13), lactate dehydrogenase (*n* = 13), total protein (*n* = 13), iron (*n* = 13), ferritin (*n* = 13), and transferrin (*n* = 12) were substituted with median values according to sex and case-control status. To rule out potential missing data bias, the overall characteristics of participants with and without missing values were compared, and no substantial differences were observed. Finally, multiple imputation of missing biomarker information was performed under the assumption of data missing at random, as previously described (23).

### Statistical analyses

Differences in baseline characteristics of HCC cases and controls were assessed by using Student’s paired *t* test, Wilcoxon’s signed-rank test, McNemar’s test, or Bowker's test of symmetry, as appropriate. Study population characteristics, including established liver cancer risk factors, were evaluated according to categories of coffee intake (<2, 2–3, 3–4, or ≥4 cups) in linear models adjusted for age, sex, and EPIC study center. (One cup was defined as 150 mL coffee.) The associations of coffee intake in categories (described above) and continuously (per cup increase) with risk of HCC were evaluated by using multivariable conditional logistic regression models, accounting for matching factors (as described above) with additional adjustment for a priori chosen covariates mainly based on previous knowledge about liver cancer risk factors. These factors included education (no school degree or primary school, secondary school, high school, or missing), smoking (never, former, current, or missing), alcohol at baseline (g/d), drinking status at baseline (nondrinker or drinker), self-reported diabetes (no, yes, or missing), HBsAg/antibodies to HCV (negative, positive, or missing), tea intake (mL/d), BMI, and waist circumference residually adjusted for BMI. To address residual confounding that may result by existing liver disease, we further adjusted the models for NAFLD, defined based on modified NAFLD noninvasive panel scoring for each of the following factors: BMI ≥28 (1 point), age at study recruitment >45 y (1 point), AST:ALT ratio ≥0.8 (1 point), self-reported diagnosis of type 2 diabetes (1 point), and serum albumin <35 g/L (1 point), with scores ≥3 considered to indicate “suspected” NAFLD (24, 25). Because risk estimates remained unchanged (<10% change in β coefficients), the variable for NAFLD was not retained in the final multivariable model. In addition, we also stratified analyses for coffee intake according to established liver cancer risk factors and tested for interactions between coffee intake and stratified variables in logistic regression models.

**FIGURE 1:**
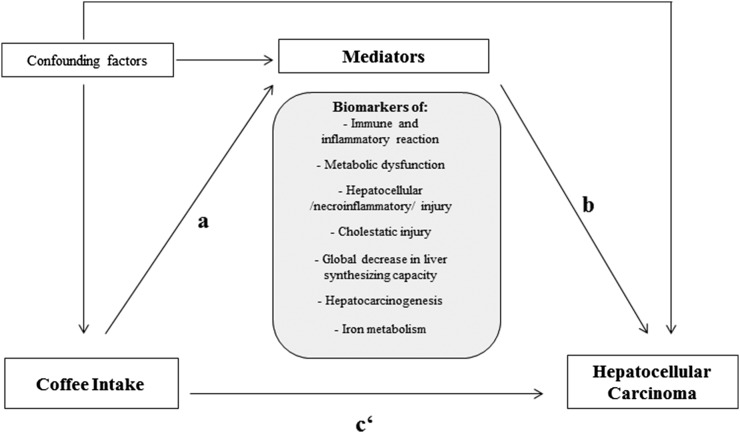
Causal diagram hypothesized for mediation and confounding, characterizing the relation between coffee intake and risk of hepatocellular carcinoma. Confounding factors represent potential factors not on the causal pathway but associated with coffee intake and hepatocellular carcinoma, including age, sex, study center, education, smoking, alcohol consumption, hepatitis B surface antigen/antibodies to hepatitis C virus infection, nonalcoholic fatty liver disease, fruit and vegetable intake, physical activity, diabetes, tea intake, and adiposity status. *a*, *b*, and *c‘* represent the paths that are evaluated at each step of mediation analysis: *a* is the association between the independent variable (coffee intake) and the mediators (biomarkers); *b* is the association between the mediator (individual biomarker levels) and the dependent variable (hepatocellular carcinoma) when controlled for the independent variable (coffee intake), assuming that *a* and *b* are statistically significant; and *c‘* is the percentage change in the association between the independent variable and dependent variable when the mediator is included in the model.

The role of each of the abovementioned biomarkers as potential mediators of the association between coffee intake and HCC risk was evaluated following published mediation principles (26). In brief, a certain variable could be considered as a mediator to the extent to which it carries the influence of a given independent variable (in our analysis “coffee intake”) on a given dependent variable (in our analysis “liver cancer”), according to the following criteria: 1) the independent variable (coffee intake) is statistically significantly associated with the potential mediator (investigated biomarker) 2), the mediator (investigated biomarker) is statistically significantly associated with the dependent variable (liver cancer) when the independent variable (coffee intake) is controlled for, and 3) the association between the independent variable (coffee intake) and the dependent variable (liver cancer) is statistically significantly attenuated on addition of the mediator (investigated biomarker) to the independent variable–dependent variable model (**Figure 1**). Note that, although the term “effect” is used in the original definitions, observational research provides estimates of RRs, and this term does not imply causality. In step 1, we evaluated the association between coffee intake (log transformed) with log-transformed concentrations of individual biomarkers in a multivariable-adjusted linear regression model (described above). In step 2, we evaluated the association between the biomarkers (per increase in log-transformed biomarker concentrations by log 2 corresponding to a doubling of concentrations on the original scale) and the risk of HCC adjusted for coffee intake by including each biomarker alternatively in the same multivariable-adjusted model. In the final step* 3* of the mediation analysis, we evaluated the associations [i.e., the regression coefficients (β coefficients) between coffee intake and liver cancer], where we estimated the percentage effect change in β coefficients in the multivariable-adjusted model and in the multivariable-adjusted model that additionally included the studied mediator. In these analyses, we modeled coffee intake as dichotomized variables (>3 compared with ≤3 cups/d) based on the visual inspection from categorical analyses showing lowered HCC risk of intakes >3 cups/d. The change in the regression coefficients was evaluated by using the difference in coefficient method, as proposed by Freedman et al (27). To evaluate the significance of the effect change, we calculated corresponding 95% CIs using Fieller’s theorem (28). In sensitivity analyses, we evaluated these associations after excluding nonfasting participants (*n* = 149) and consumers of very low (≤30 mL/d) and very high (>1500 g/d) amounts of coffee (8 cases and 9 controls). We also repeated the mediation analyses after excluding cases with a diagnosis of HCC within the first 2 y of study follow-up (*n* = 24) and in a subset of participants with complete biomarker information. Two-sided *P* values <0.05 were considered to indicate statistical significance. All statistical analyses were performed by using SAS version 9.2 (SAS Institute Inc.).

## RESULTS

### Descriptive analyses

The baseline characteristics of incident HCC cases and their corresponding controls are presented in **Table 1**. As compared with the controls, cases with HCC had lower intakes of fruit and vegetables. They were also more likely to be smokers, to be high alcohol consumers, to be patients with diabetes, and to be positive for HBsAg/antibodies to HCV infection. Compared with controls, HCC cases had statistically significantly higher BMIs, waist circumferences, and concentrations of the investigated biomarkers, except for albumin (inversely associated) and transferrin (not significantly associated). In **Table 2**, the age- and sex-adjusted study population characteristics and established HCC risk factors according to categories of coffee intakes in healthy controls are shown. Increasing intakes of coffee were positively associated with a university degree, smoking, and alcohol intake and inversely associated with female sex, age, intake of fruit and vegetables, BMI, waist circumference, and waist-to-hip ratio.

**TABLE 1:** Baseline characteristics of hepatocellular carcinoma cases and controls: EPIC^1^

	Hepatocellular carcinoma		
Variables	Cases (*n* = 125)	Controls (*n* = 250)	P^2^
Female sex,^3^ %	32.0	32.0	0.31
Age,^3^ y	60.1 ± 6.6^4^	60.1 ± 6.6	0.43
University degree, %	16.0	19.6	0.06
Physically inactive, %	8.8	13.6	0.04
Smoker, %	38.4	18.8	<0.0001
Coffee intake, mL/d	394.2 ± 440.7	448.0 ± 431.1	0.06
Tea intake, mL/d	124.3 ± 271.5	151.1 ± 273.3	0.16
Alcohol intake, g/d	20.8 ± 33.1	15.4 ± 19.6	0.01
Patients with diabetes, %	12.8	6.4	0.03
Fruit intake, g/d	211.8 ± 167.0	238.1 ± 208.8	0.04
Vegetable intake, g/d	171.4 ± 133.2	193.8 ± 142.9	0.007
Total energy intake, kcal/d	2144 ± 639.4	2199 ± 574.9	0.24
Anthropometric factors			
BMI, kg/m^2^	28.1 ± 5.27	27.0 ± 3.9	0.003
Waist circumference, cm	97.1 ± 15.1	92.6 ± 11.2	<0.0001
Waist-to-hip ratio	0.93 ± 0.09	0.91 ± 0.08	<0.0001
NAFLD,^5^ %	48.3	29.2	<0.02
Hepatitis infection (HBsAg + HCVAg) seropositivity, %	10.7	3.5	<0.0001
Biomarkers			
Immune and inflammatory reaction			
CRP, mg/L	4.89 ± 9.06	2.03 ± 2.65	<0.0001
IL-6, pg/mL	5.86 ± 12.03	2.11 ± 1.69	0.0002
Metabolic dysfunction			
C-peptide, ng/mL	3.89 ± 2.65	2.62 ± 1.74	0.005
Fetuin A, μg/mL	213.0 ± 50.1	202.8 ± 42.2	0.008
Adiponectin, μg/mL	6.5 ± 4.2	5.3 ± 2.7	<0.0001
Leptin, ng/mL	12.9 ± 11.7	10.4 ± 10.2	0.003
Hepatocellular /necroinflammatory/ injury			
GLDH, μmol · s^−1^ · L^−1^	165.0 ± 154.5	90.2 ± 101.2	<0.0001
ALT, U/L	46.0 ± 41.0	20.9 ± 14.1	<0.0001
AST, U/L	52.7 ± 44.2	20.9 ± 9.4	<0.0001
LDH, U/L	170.2 ± 37.9	155.8 ± 36.9	<0.0001
Cholestatic injury			
GGT, U/L	167.3 ± 248.8	33.1 ± 41.1	<0.0001
ALP, U/L	99.9 ± 77.2	63.2 ± 18.4	<0.0001
Global decrease in liver synthesizing capacity			
Albumin, g/L	39.1 ± 4.4	42.1 ± 3.3	<0.0001
Total bilirubin, μmol/L	12.9 ± 11.1	8.4 ± 4.2	<0.0001
Total protein, g/L	72.7 ± 6.1	71.0 ± 5.5	<0.0001
Hepatocarcinogenesis			
AFP, kUI/L	267.0 ± 179.3	3.85 ± 2.52	<0.0001
Iron metabolism			
Iron, μmol/L	21.5 ± 8.9	18.4 ± 5.8	<0.0001
Ferritin, μmol/L	323.8 ± 56.0	156.2 ± 166.8	<0.0001
Transferrin, mg/mL	2.42 ± 0.42	2.40 ± 0.30	0.74

1 AFP, α-fetoprotein; ALP, alkaline phosphatase; ALT, alanine aminotransferase; AST, aspartate aminotransferase; CRP, C-reactive protein; EPIC, European Prospective Investigation into Cancer and Nutrition; GGT, γ-glutamyltransferase; GLDH, glutamate dehydrogenase; HBsAg, hepatitis B surface antigen; HCVAg, hepatitis C virus antigen; LDH, lactatate dehydrogenase; NAFLD, nonalcoholic fatty liver disease.

2 Case-control differences were assessed by using Student’s paired *t* test, Wilcoxon’s signed-rank test, McNemar’s test, or Bowker's test of symmetry, where appropriate.

3 Sex and age at recruitment were among the matching criteria.

4 Mean ± SD (all such values).

5 NAFLD was defined by using modified NAFLD diagnostic panel scoring for each of the following factors: BMI (in kg/m^2^) ≥28 (1 point), age at study recruitment >45 y (1 point), AST:ALT ratio ≥0.8 (1 point), reported diagnosis of type 2 diabetes (1 point), and serum albumin <35 g/L (1 point). Scores ≥3 were considered to indicate NAFLD (24, 25).

**TABLE 2:** Age- and sex-adjusted characteristics, by quintiles of coffee intake in the control study population (*n* = 250): EPIC^1^

	Coffee intake^2^				
Variables	≤2 cups/d (ref)	2 to ≤3 cups/d	3 to ≤4 cups/d	>4 cups/d	*P*-trend^3^
Female sex, %	34	49	23	24	0.05
Age, y	61	60.7	60.4	58.5	0.01
University degree, %	14	14	18	20	0.01
Physically inactive, %	12	14	15	16	0.10
Smoker, %	15	9	14	32	0.002
Alcohol intake, g/d	14	9	15	19	0.02
Patients with diabetes, %	15	5	19	30	0.15
Hepatitis infection (HBsAg + HCVAg) seropositivity, %	9	23	11	18	0.05
Suspected NAFLD, %	33	32	24	33	0.66
Fruit intake, g/d	306.6	219.2	199.7	165.4	0.0006
Vegetable intake, g/d	216.7	172.9	144.8	167.7	0.01
Total energy intake, kcal/d	2133.1	2022.2	2149.7	2136.7	0.19
BMI, kg/m^2^	27.6	26.1	26.8	26.6	0.02
Waist circumference, cm	91.5	88.2	90.5	89.9	0.02
Waist-to-hip ratio	0.89	0.87	0.88	0.87	0.03

1 EPIC, European Prospective Investigation into Cancer and Nutrition; HBsAg, hepatitis B surface antigen; HCVAg, hepatitis C virus antigen; NAFLD, nonalcoholic fatty liver disease.

2 1 cup = 250 mL.

3 Derived from a linear model calculated by using the median intakes of coffee within categories as continuous variables adjusted for age at study recruitment, sex, and study center.

### Association of coffee intake with HCC risk and biomarkers

**FIGURE 2:**
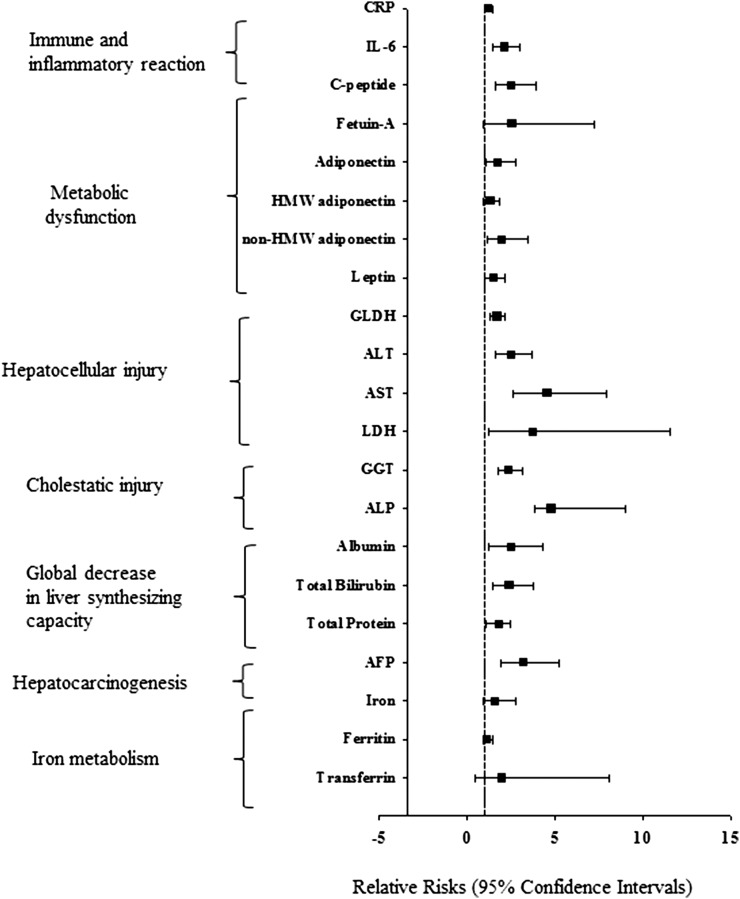
Association of individual biomarkers (log transformed) with risk of hepatocellular carcinoma in a multivariable model adjusted for coffee intake (mL/d) in the European Prospective Investigation into Cancer and Nutrition (EPIC). The multivariable model in conditional logistic regression accounted for the following matching factors: age, sex, study center, follow-up time since blood collection, time of day at blood collection, and fasting status plus adjustment for education (no school degree or primary school, technical or professional school, secondary school, university degree, or unknown), smoking status (never, past, current, or unknown), alcohol intake (mL/d; continuous), nondrinking (categorical), hepatitis B surface antigen/antibodies to hepatitis C virus infection (positive, negative, or unknown), fruit and vegetable intake (g/d; continuous), physical activity (inactive, moderately inactive, moderately active, active, or missing), diabetes (yes, no, or missing), tea intake (mL/d), BMI (in kg/m^2^; continuous), and waist circumference adjusted for BMI by using the residual method (cm; continuous). Women were further matched by menopausal status and phase of menstrual cycle at blood collection; postmenopausal women were matched on use of hormone replacement therapy. The associations between metabolic biomarkers and risk of hepatocellular carcinoma in EPIC were originally reported by Aleksandrova et al. (20). AFP, α-fetoprotein; ALP, alkaline phosphatase; ALT, alanine aminotransferase; AST, aspartate aminotransferase; CRP, C-reactive protein; GGT, γ-glutamyltransferase; GLDH, glutamate dehydrogenase; HMW, high molecular weight; LDH, lactate dehydrogenase.

In a multivariable-adjusted model including established HCC risk factors, coffee intake was associated with a lower risk of HCC, RR per 1 cup/d: 0.87 (95% CI: 0.77, 0.98). For participants with an intake of ≥4 cups coffee/d compared with those with <2 cups/d, the multivariable-adjusted RR was 0.25 (95% CI: 0.11, 0.62; *P*-trend = 0.006). The associations of coffee intake with all potential mediators (selected biomarkers) are shown in **Table 3**. After control for case-control status and established liver cancer risk factors, coffee intake was positively associated with C-peptide and inversely with IL-6, GLDH, ALT, AST, GGT, alkaline phosphatase, total bilirubin, and AFP. The RRs for HCC in relation to the selected biomarkers, as estimated by including each biomarker in the multivariable-adjusted model, are shown in **Figure 2**. Each of the biomarkers was associated with a higher risk of HCC, with the exception of transferrin, which was consequently excluded from the mediation analysis.

**TABLE 3:** Multivariable-adjusted linear regression models for the association between coffee intake and biomarker concentrations: EPIC^1^

Biomarkers	Multivariable-adjusted β coefficient (95% CI)^2^	*P *
Immune and inflammatory reaction		
CRP, mg/L	−0.11 (−0.22, 0.00)	0.06
IL-6, pg/mL	−0.18 (−0.35, −0.02)	0.04
Metabolic dysfunction		
C-peptide, ng/mL	0.25 (0.03, 0.48)	0.02
Fetuin A, μg/mL	−0.02 (−0.62, 0.57)	0.94
Adiponectin, μg/mL	−0.15 (−0.46, 0.10)	0.35
HMW adiponectin, μg/mL	−0.06 (−0.28, 0.15)	0.59
Non-HMW adiponectin, μg/mL	−0.37 (−0.72, −0.03)	0.03
Leptin, ng/mL	0.14 (−0.02, 0.30)	0.08
Hepatocellular/necroinflammatory/injury		
GLDH, μmol · s^−1^ · L^−1^	−0.19 (−0.36, −0.01)	0.04
ALT, U/L	−0.22 (−0.38, −0.02)	0.04
AST, U/L	−0.20 (−0.49, 0.00)	0.02
LDH, U/L	−0.15 (−0.76, 0.44)	0.60
Cholestatic injury		
GGT, U/L	−0.20 (−0.37, −0.04)	0.01
ALP, U/L	−0.45 (−0.83, −0.08)	0.01
Global decrease in liver synthesizing capacity		
Albumin, g/L	0.86 (−0.56, 2.30)	0.23
Total bilirubin, μmol/L	−0.30 (−0.55, −0.05)	0.02
Total protein, g/L	−0.99 (−2.65, 0.67)	0.24
Hepatocarcinogenesis		
AFP, kUI/L	−0.15 (−0.26, −0.01)	0.03
Iron metabolism		
Iron, μmol/L	0.28 (−0.03, 0.68)	0.11
Ferritin, μmol/L	0.04 (−0.10, 0.18)	0.55
Transferrin, mg/mL	−0.28 (−1.17, 0.59)	0.52

1 The multivariable models (based on log-transformed variables of coffee intake and log-transformed biomarker variables) accounted for case-control status and matching factors: age (reported at study recruitment), sex, study center, follow-up time since blood collection, time of day of blood collection, and fasting status plus adjustment for education (no school degree or primary school, technical or professional school, secondary school, university degree, or unknown), smoking status (never, past, current, or unknown), alcohol intake (mL/d; continuous), nondrinking (categorical), hepatitis B surface antigen/antibodies to hepatitis C virus infection (positive, negative, or unknown), fruit and vegetable intake (g/d; continuous), physical activity (inactive, moderately inactive, moderately active, active, or missing), diabetes (yes, no, or missing), tea intake (mL/d), BMI (in kg/m^2^; continuous), and waist circumference adjusted for BMI by using the residual method (cm; continuous). Women were further matched by menopausal status and phase of menstrual cycle at blood collection; postmenopausal women were matched on use of hormone replacement therapy. Nonconsumers of coffee (11 cases/12 controls) were not included in these analyses. AFP, α-fetoprotein; ALP, alkaline phosphatase; ALT, alanine aminotransferase; AST, aspartate aminotransferase; CRP, C-reactive protein; EPIC, European Prospective Investigation into Cancer and Nutrition; GGT, γ-glutamyltransferase; GLDH, glutamate dehydrogenase; HMW, high molecular weight; LDH, lactatate dehydrogenase.

2 Estimated in multivariable-adjusted linear regression models (*P* values).

#### Mediation analyses

**Table 4** shows the mediating effects of each of the biomarkers deemed statistically significant in the previous analyses, expressed as the percentage change (and associated 95% CIs) in the estimated log of the association (β coefficient) of coffee intake on HCC risk. A statistically significant attenuation of the association of coffee with HCC risk was observed for IL-6, GLDH, ALT, AST, GGT, and total bilirubin, which in combination attenuated the β coefficients by 72% (95% CI: 7%, 239%). Of these, IL-6, AST, and GGT produced the highest change in the regression coefficients: 40%, 56%, and 60%, respectively (Table 4).

**TABLE 4:** RRs, 95% CIs, and regression coefficients for risk of hepatocellular carcinoma associated with coffee intake (>3 compared with ≤3 cups/d) and percentage change in regression coefficients with adjustment for individual biomarkers: EPIC^1^

Adjustment for biomarkers	RR (95% CI)	β Coefficient^2^	Effect change^3^ (95% CI),^4^ %
Multivariable model	0.35 (0.17, 0.76)	−1.03	
Multivariable model with additional adjustment for individual biomarkers			
* *Immune and inflammatory reaction			
CRP, mg/L	0.37 (0.16, 0.83)	−0.99	4 (−15, 42)
IL-6, pg/mL	0.53 (0.23, 1.23)	−0.62	40 (7, 125)
* *Metabolic dysfunction			
C-peptide, ng/mL	0.37 (0.16, 0.84)	−0.99	4 (−27, 45)
Fetuin A, μg/mL	0.36 (0.17, 0.79)	−1.01	2 (−14, 17)
Adiponectin, μg/mL	0.37 (0.16, 0.80)	−0.99	3 (−13, 25)
HMW adiponectin, μg/mL	0.39 (0.18, 0.86)	−0.93	3 (−12, 20)
Non-HMW adiponectin, μg/mL	0.42 (0.19, 0.92)	−0.87	8 (−11, 41)
Leptin, ng/mL	0.37 (0.17, 0.82)	−0.98	4 (−16, 34)
* *Hepatocellular/necroinflammatory/injury			
GLDH, μmol · s^−1^ · L^−1^	0.45 (0.19, 1.08)	−0.77	24 (0, 102)
ALT, U/L	0.46 (0.19, 1.05)	−0.78	24 (0, 84)
AST, U/L	0.63 (0.25, 1.61)	−0.45	56 (9, 182)
LDH, U/L	0.42 (0.19, 0.93)	−0.86	17 (−5, 65)
Cholestatic injury			
GGT, U/L	0.66 (0.25, 1.78)	−0.40	60 (7, 190)
ALP, U/L	0.37 (0.14, 0.94)	−1.00	3 (−57, 71)
Global decrease in liver synthesizing capacity			
Albumin, g/L	0.39 (0.16, 0.94)	−0.92	10 (−16, 61)
Total bilirubin, μmol/L	0.45 (0.19, 1.04)	−0.79	23 (2, 72)
Total protein, g/L	0.40 (0.18, 0.88)	−0.90	12 (−3, 47)
Hepatocarcinogenesis			
AFP, kUI/L	0.45 (0.20, 1.05)	−0.79	23 (−2, 81)
Iron metabolism^5^			
Iron, μmol/L	0.30 (0.13, 0.67)	−1.19	−15 (−64, 7)
Ferritin, μmol/L	0.33 (0.15, 0.75)	−1.08	−4 (−33, 18)

1 The multivariable model accounted for matching factors: age, sex, study center, follow-up time since blood collection, time of day of blood collection, and fasting status plus adjustment for education (no school degree or primary school, technical or professional school, secondary school, university degree, or unknown), smoking status (never, past, current, or unknown), alcohol intake (mL/d; continuous), nondrinking (categorical), hepatitis B surface antigen/antibodies to hepatitis C virus infection (positive, negative, or unknown), fruit and vegetable intake (g/d; continuous), physical activity (inactive, moderately inactive, moderately active, active, or missing), diabetes (yes, no, or missing), tea intake (mL/d), BMI (in kg/m^2^; continuous), and waist circumference adjusted for BMI by using the residual method (cm; continuous). Women were further matched by menopausal status and phase of menstrual cycle at blood collection; postmenopausal women were matched on use of hormone replacement therapy. 1 cup = 250 mL. AFP, α-fetoprotein; ALP, alkaline phosphatase; ALT, alanine aminotransferase; AST, aspartate aminotransferase; CRP, C-reactive protein; EPIC, European Prospective Investigation into Cancer and Nutrition; GGT, γ-glutamyltransferase; GLDH, glutamate dehydrogenase; HMW, high molecular weight; LDH, lactatate dehydrogenase.

2 The β coefficient (regression coefficient) is the natural log of the RR estimate.

3 The percentage change in the regression coefficient with adjustment for each additional biomarker compared with the multivariable model.

4 The 95% CI was calculated based on Fieller’s theorem (28).

5 The biomarker of iron metabolism, transferrin, was excluded from the analysis because it was not associated with coffee intake or risk of hepatocellular carcinoma and therefore did not meet the criteria for being a potential mediator.

#### Stratified and sensitivity analyses

Sex was not a statistically significant effect modifier in the associations of coffee and HCC risk (*P*-interaction by sex = 0.23). Nevertheless, in stratified analyses, the associations between coffee intake and HCC risk proved to be statistically significant in men (RR_>3 vs. ≤3 cups/d_ = 0.33; 95% CI: 0.13, 0.78) but not in women (RR_>3 vs. ≤3 cups/d_ = 0.89; 95% CI: 0.22, 3.60). When the analysis was stratified according to categories of non-NAFLD, and according to other established liver cancer risk factors, no statistically significant interaction was observed by any of these factors (**Supplemental Table 1**). Because of the small number of cases in the stratified analyses, these results should be interpreted with caution. In sensitivity analyses that excluded cases in the first 2 y of study, follow-up did not appreciably alter the risk estimates (RR _>3 vs. ≤3 cups/d_ = 0.43; 95% CI: 0.19, 0.97). After exclusion of participants with nonfasting biomarker samples and those with very low and very high intakes of coffee, the main results remained generally unaltered. An exception was the altered association with C-peptide, which was no longer statistically significant after the exclusion of nonfasting participants (β = −0.11, *P* = 0.78). Finally, when the analyses were repeated only in participants with complete biomarker data or in a data set generated based on multiple imputation of missing biomarker data, the results remained unchanged (data not shown).

## DISCUSSION

In this prospective nested case-control study within the large EPIC cohort, we evaluated the potential mediating effects of inflammatory, metabolic, liver injury, and iron metabolism biomarkers on the inverse association of coffee intake with HCC risk. Our data suggest that this association is mostly accounted for by IL-6 as a biomarker of inflammation and innate immunity and by biomarkers of hepatocellular and cholestatic injury: GLDH, ALT, AST, GGT, and bilirubin. To our knowledge, this was the first attempt to uncover possible mediating biomarkers of the association between coffee intake and HCC risk in a prospective study setting. Notably, although we observed an inverse association between coffee intake and several biomarkers in line with prior evidence, only few of those biomarkers proved to be mediators of the coffee–liver cancer association.

IL-6 has been known as a pleiotropic cytokine produced by the liver during the acute phase response active in immune regulation, inflammation and oncogenesis (29). Previously, in EPIC, we reported on the positive association between IL-6 and HCC independent of adiposity and metabolic biomarkers, which suggests a role for innate immunity in liver cancer pathogenesis (20). High consumption of coffee has been suggested to exert immune-boosting effects on NAFLD independent of potential antioxidant effects (32).The anti-inflammatory properties of coffee were also reported among high-risk individuals in both experimental (30) and observational (31) studies. Of note, our analyses suggest that these effects are specific to IL-6 and were not shown for CRP, another inflammatory biomarker included in our analysis. However, inverse associations between coffee intake and CRP concentrations have been previously suggested (33). Therefore, further detailed studies are needed to shed more light on these associations.

The potential hepatoprotective effects of coffee may represent another main mechanism behind its inverse relation with HCC risk. Epidemiological studies have shown associations between abnormally high liver enzyme concentrations and cancer incidence and mortality (34, 35). Coffee consumption has been consistently associated with improved serum enzyme concentrations in a dose-dependent manner (36). In particular, our data point to the role of AST and GGT as the 2 biomarkers that explain the highest proportion of the inverse association between coffee intake and HCC risk. In clinical practice, augmentation in AST concentrations has been used to indicate the presence of hepatocellular-predominant disorders, whereas increases in GGT have been related to cholestatic-predominant diseases (37); therefore, coffee seems to be implicated in both of these pathologies. In addition, AST and GGT have been related to inflammation and oxidative stress. Thus, AST acts as an important mediator of inflammatory processes and nonspecific liver injury. Elevated AST concentrations have been reported in diseases that affect organs other than the liver, such as myocardial infarction, acute pancreatitis, acute renal disease, and musculoskeletal diseases. GGT is used as a sensitive indicator of hepatic inflammation, fatty liver disease, and hepatitis; most recently, it has been implicated in oxidative stress associated with glutathione metabolism (38). Interestingly, our data show that a high coffee intake is associated with lower concentrations of a liver-specific mitochondrial enzyme—GLDH—and, thereby, with a lower risk of HCC. GLDH is particularly indicative of toxic parenchymal liver injury; thereby, our findings may add to previous evidence showing that coffee is associated with liver damage progression irrespective of the etiology (39).

It has been largely speculated that the inverse association between coffee intake and liver cancer could be accounted for by reverse causation bias in epidemiological studies because of the inclusion of participants with underlying liver disease who reduce coffee consumption as a result of physician recommendations (3). However, protective effects of coffee have also been reported in advanced disease states. In particular, beneficial associations of coffee intake have been reported in a variety of populations at higher risk of liver diseases, including those with excessive alcohol intake, who are obese, who are smokers, and with chronic viral hepatitis (40). Previous prospective cohort studies observed that coffee drinkers were more likely to respond to HCV therapy (43) and less likely to progress toward end-stage liver disease, including liver cancer (41). Furthermore, among chronic hepatitis B virus carriers, patients who reported coffee drinking of ≥4 times/wk had a 59% lower risk of having liver cancer than did those who reported abstaining from coffee drinking (42). Reported associations were independent of both clinical and histological markers of underlying liver disease, which argues against speculated reverse causality of the observed associations between coffee and liver cancer.

In our study we specifically accounted for reverse causation by evaluating potential effect modification by NAFLD, alcohol consumption, and smoking as important independent risk factors for HCC in populations with a low prevalence of hepatitis infection (21). However, we did not detect differences in the results by any of the studied factors; therefore, it is seems unlikely that our main findings could have been influenced by possible physicians’ recommendation and drinking restrictions. Furthermore, we observed that participants with a higher coffee intake were more likely to practice other unhealthy behaviors, such as smoking, higher alcohol consumption (particularly in women), and lower intakes of fruit and vegetables. Therefore, it is also unlikely that suggested favorable effects of coffee could be explained by other healthy behaviors, such as lower alcohol drinking, in (heavy) coffee consumers.

The suggested anti-inflammatory and hepatoprotective effects of coffee in our study could be accounted for by several bioactive compounds with high antioxidant capacity. The main compounds in coffee implicated to have protective roles in the liver are caffeine, paraxanthine, cafestol, kahweol, and chlorogenic acids; however, >1000 additional compounds could be responsible for its beneficial effects (43, 44, 45). Additional studies are warranted to evaluate the potential for application of these specific biochemical compounds in HCC prevention.

The strengths of our study included its prospective design, the exploration of a wide range of biomarkers representative of different pathophysiological processes, and the detailed information on several dietary, lifestyle and anthropometric factors available to adjust for potential confounders in the analyses. Some limitations of the current study should also be considered. We used a common variable for the assessment of coffee intake and did not differentiate between different kinds of coffee (caffeinated compared with decaffeinated; filtered compared with not filtered). Our data also do not provide information on potential active compounds that may be particularly relevant to liver protection. Despite controlling our analysis for several factors, we cannot completely rule out the potential of residual confounding by unmeasured factors. For example, we did not have information on genetic variants (such as CYP1A2) that are known to exert the ability to modify caffeine metabolism and consequently potentially responsible for the amount of coffee consumed. Furthermore, the biomarkers used in the analyses were assessed at a single point in time at study baseline and may be susceptible to short-term variation, which could lead to bias toward the null. However, previously, most of the biomarkers indicated the high reliability of single measurements over time (22). Not all of the participants were fasting at the time of blood draw, which limited the analysis on biomarkers—the levels of which are dependent on fasting status. Therefore, we have reported findings after excluding nonfasting participants. Because some of the biomarkers included in the analysis are interrelated, we cannot exclude the possibility that we may have partially accounted for the effect of certain other related biomarkers when adjusting for one biomarker. Finally, these data should still be cautiously interpreted because the suggested mediators may simply be statistical intermediates and/or markers of various pathogenic processes not essentially on the causal pathway to HCC. Furthermore, caution should be paid to the fact that, irrespective of the different methods used to account for reverse causality in our data and the biologically plausibility behind observed biomarker effects, we cannot completely exclude the possibility that low coffee intake may be a marker of poor liver health and/or pre-existing disease.

In conclusion, the association of coffee intake with HCC risk in this large European cohort study was statistically accounted for by biomarkers of inflammation and hepatocellular injury. Because of difficulties in conducting long-term randomized trials to test these relations, our findings may provide important insights into the current knowledge on the prevention of HCC—one of the most lethal tumors in the world.

## Footnotes

48 Abbreviations used: AFP, α-fetoprotein; ALT, alanine aminotransferase; AST, aspartate aminotransferase; CRP, C-reactive protein; EPIC, European Prospective Investigation into Cancer and Nutrition; GGT, γ-glutamyltransferase; GLDH, glutamate dehydrogenase; HBsAg, hepatitis B surface antigen; HCC, hepatocellular carcinoma; HCV, hepatitis C virus; HMW, high molecular weight; NAFLD, nonalcoholic fatty liver disease.
